# The psychometric properties and clinical utility of neural measures of reward processing

**DOI:** 10.1093/scan/nsad007

**Published:** 2023-03-15

**Authors:** Nader Amir, Amanda Holbrook, Emily Meissel, William Taboas

**Affiliations:** Department of Psychology, Joint Doctoral Program at San Diego State University/University of California in San Diego, San Diego 92120, USA; Department of Psychology, Joint Doctoral Program at San Diego State University/University of California in San Diego, San Diego 92120, USA; Department of Psychology, Joint Doctoral Program at San Diego State University/University of California in San Diego, San Diego 92120, USA; Department of Psychology, Joint Doctoral Program at San Diego State University/University of California in San Diego, San Diego 92120, USA

**Keywords:** psychometric properties, reliability, validity, RewP, FN, reward processing

## Abstract

Reward processing is implicated in the etiology of several psychological conditions including depressive disorders. In the current paper, we examined the psychometric properties of a neural measure of reward processing, the reward positivity (RewP), in 279 adult women at baseline and 187 women 8 weeks later. The RewP demonstrated excellent internal consistency at both timepoints and good test–retest reliability using estimates from both classical test theory and generalizability theory. Additionally, the difference between RewP following reward and loss feedback was marginally associated with depressive symptoms in a subsample of participants. We also examined the relationship between subject-level dependability estimates and depression severity, finding that depressive symptoms may contribute to lower dependability on reward trials. However, this finding did not survive correction for multiple comparisons and should be replicated in future studies. These findings support RewP as a useful measure of individual differences of reward processing and point to the potential utility of this measure for various forms of psychopathology.

## Introduction

Abnormalities in reward processes may be central to the etiology of different clinical conditions including substance abuse (Hixsona *et al.*, 2019), eating disorders (Simon *et al.*, 2016), and depressive disorders (Henriques *et al.*, 1994; Henriques and Davidson, 2000). Researchers have relied on behavioral and self-report measures (Kasch *et al.*, 2002; Pizzagalli *et al.*, 2008; Whitton *et al.*, 2015), as well as neuroimaging techniques (e.g. functional magnetic resonance imaging; fMRI), to examine reward processing. For example, individuals with major depressive disorder exhibit reduced activation in the ventral striatum, a brain region implicated in reward processing (Forbes *et al.*, 2009; Pizzagalli *et al.*, 2009). To examine the time course of reward processing, researchers have used the reward positivity (RewP), a positive deflection in the event-related potential (ERP^1^) component of the electroencephalogram (EEG) approximately 250–350 ms after the presentation of a reward (for review, see Proudfit *et al.*, 2015). Neural reactivity after rewards (RewP-gain) and after loss (RewP-loss) and the difference between the two (ΔRewP) are typically assessed using a guessing task such as the doors task (Dunning and Hajcak, 2007; Foti and Hajcak, 2009, 2010). In the doors task, participants see two identical images of doors on a computer screen and select one of them by pressing a mouse button. The selection will result in either a monetary reward (e.g. $0.50) or a monetary loss (e.g. $0.25), thereby generating a RewP-gain or a RewP-loss, respectively, approximately 250–350 ms after the presentation of the feedback. The doors task is the most frequently used method of eliciting RewP measures across developmental stages and in clinical samples, in part due to the low participant burden (typically <7 min). Thus, we focus on the doors task as a measure of reward sensitivity in the current paper.

A blunted ΔRewP while completing the doors task has been associated with increased depressive symptoms in children, adolescents (Bress *et al.*, 2015; Belden *et al.*, 2016), and adults (Foti and Hajcak, 2009; Funkhouser *et al.*, 2021). Moreover, the ΔRewP is related to risk for depression. For example, a blunted ΔRewP may be a risk factor for depression in never-depressed children and adolescents (Kujawa *et al.*, 2018; Nelson and Jarcho, 2021) and interact with other prominent risk factors (e.g. maternal suicidality) to predict increases in depression in children (Burani *et al.*, 2021). Finally, ΔRewP can predict remission status and successful response to treatment in depressed adults (Klawohn *et al.*, 2021) as well as change in depressive symptoms in anxious children and adolescents following treatment (Kujawa *et al.*, 2018). Thus, these studies suggest that ΔRewP can predict the onset and course of depression at the individual level. However, in order to incorporate neural markers of reward sensitivity such as ΔRewP into diagnostic classification and examine mechanism of change as a result of gold standard treatments, it is essential to ensure adequate psychometric properties of these measures at the individual level (Hajcak *et al.*, 2019).

Examining this question in late childhood and adolescence, Bress *et al.* (2015), Luking *et al.* (2017), and Kujawa *et al.* (2018) found that split-half estimates of internal consistency were high for RewP-gain and RewP-loss (*r*’s > 0.79) but not ΔRewP (*r*’s = 0.28–0.50). Similarly, RewP-gain and RewP-loss demonstrated acceptable test–retest reliability (*r*’s = 0.52–0.67) across 2-to-3 years. However, ΔRewP showed low-to-moderate test–retest reliability (*r*’s = 0.18–0.43).

Examining this question in adult populations, Levinson *et al.* (2017) and Distefano *et al.* (2018) found that RewP-gain and RewP-loss showed high internal consistency (split-half, Cronbach’s alpha, dependability; 0.71–0.91) but the ΔRewP did not (*a*’s = 0.28–0.45). Moreover, RewP-gain showed good 1-week test–retest reliability (*r* = 0.71), while RewP-loss (*r* = 0.45) and ΔRewP (*r* = 0.27) showed moderate-to-low test–retest reliability, respectively (Levinson *et al.*, 2017). Finally, in one of the largest studies examining the internal consistency of RewP (split-half, Cronbach’s alpha, and dependability) in participants aged 10 to 55, Ethridge and Weinberg (2018) found that RewP-gain and RewP-loss had excellent (0.86–0.93) internal consistency, but residual and subtraction-based ΔRewP scores demonstrated more variable and overall weaker internal consistency (0.43–0.85). To examine whether internal consistency varied by age, these researchers divided their sample into three age groups (adolescence: ages 10–17, *n* = 27; early adulthood: ages 18–24, *n* = 182; and middle adulthood: ages 33–55, *n* = 31). Age did not significantly moderate the split-half reliability of any reliability components. However, a larger number of trials were required to reach an acceptable internal consistency in the adolescent and middle adult groups, as compared to early adults. These researchers concluded that this age difference in the number of trials needed may be in part due to differences in sample size in each age group. Indeed, most of the participants in this study as well as other adult studies (Levinson *et al.*, 2017; Distefano *et al.*, 2018) comprised young adults (age 18–24).

In summary, similarities across youth and adult samples suggest test–retest reliability, and internal consistency of RewP-gain and RewP-loss are moderate to excellent, whereas ΔRewP reliability is typically low to moderate. Lower reliability of difference scores is common across areas of research due to highly correlated constituent scores (Clayson *et al.*, 2021a). Moreover, difference scores are affected by noise and measurement error found in both constituent scores (Furr and Bacharach, 2014), thus restricting the amount of true variance.

Thus, four questions remain regarding the psychometric properties of the RewP. First, few studies have examined the psychometric properties of RewP in an adult samples aged 24 and older. Indeed, across various studies, only Ethridge and Weinberg (2018) included adults aged 24 and older and their sample comprised 31 individuals. Depressive disorders begin to increase in prevalence in those aged 20 to 30 and continue to increase into middle age, with the highest rates of depression reported among those aged 40–59 (Centers for Disease Control and Prevention (CDC), 2010; Pratt and Brody, 2014). Moreover, incidence of depression is higher among women in this age group. Thus, it important to establish the reliability of RewP and its relationship with depression in a sample of middle-aged women.

Second, previous studies have focused on reporting group-level reliability estimates (i.e. a single reliability score for the entire group) of RewP measures. It is possible that this single score can mask low reliability or data quality of some participants. Here we consider two different measures of subject-level data quality: (i) standardized measurement error (SME) and (ii) subject-level dependability.

Luck *et al.* (2021) recommended the computation of SME as an estimate of individual-level data quality. The SME is computed for time window (i.e. average ERP activity between 250 and 350 ms following feedback) separately for each condition (i.e. gain and loss) and for each participant. Briefly, the SME*_ij_* for trial scores, *i*, from a given person, *j*, is estimated by calculating the standard deviation of the single-trial scores for a given person (σ*_ij_*) and dividing by the square root of the number of trials (*n_ij_*). The SME*_ij_* quantifies the data quality for each individual participant, making it possible to identify participants with ‘low quality’ data relative to the rest of the sample, such that higher SME*_ij_* scores reflect greater measurement error than lower SME*_ij_* scores. SME*_ij_* scores are trial-dependent such that a participant with few trials will have a larger SME than a participant with many trials when between-trial standard deviations (σ*_ij_*) for the two participants are identical. SME*_ij_* scores provide no information on whether between-trial variance is small compared to between-person variance. Therefore, a person’s data could have high internal consistency in one group but low internal consistency in another group, despite the fact that SME*_ij_* would be identical.

Subject-level dependability quantifies whether person-specific data quality is high enough for the examination of individual differences within a specific group (Clayson *et al.*, 2021b). Briefly, subject-level dependability (ϕ*_jk_*) for a given person, *j*, from a group, *k*, is calculated as a function of between-person variance (σ^2^*_p_*), person-specific between-trial variance (σ^2^*_ijk_*), and the person-specific number of trials (*n_ijk_*). Conceptually, ϕ*_jk_* is the ratio comparing the size of between-person differences in average ERP scores from a group to the variability of single-trial ERP scores that contribute of an individual’s average ERP score. A benefit of ϕ*_jk_* is that it uses an approximation of data quality estimates in its calculation and scales it using between-person variance. Thus, ϕ*_jk_* is conceptually an estimate of data quality for an intended purpose (e.g. is data quality high enough to examine individual differences in the current sample?). The interpretation of ϕ*_jk_* is similar to that of group-level estimates ranging between 0 and 1, with estimates closer to 1 indicating higher internal consistency (i.e. dependability). Scores with high internal consistency (e.g. *>*0.80) are well suited for examining individual differences between participants (Clayson *et al.*, 2021b).

Third, although previous ERP studies have shown subject-level reliability varies within a sample (Clayson *et al.*, 2021b), it is unclear what factors (e.g. demographic and psychiatric measures) may contribute to lower reliability of RewP in some individuals. For example, while Distefano *et al.* (2018) suggested differences in sample size may be one explanation for why adolescents and middle-aged adults required a larger number of trials to reach acceptable internal consistency in their study, it is also possible that data quality and reliability varied as a function of age, in turn requiring a larger number of trials needed to achieve a reliable RewP in certain age groups. This may be a particularly important consideration for studying RewP in developmental studies when change in RewP measures is assessed multiple times across different ages. It is also possible that psychiatric symptoms (e.g. depression) may be uniquely related to subject-level variability of RewP. While previous EEG and fMRI studies have found that individuals with autism, attention-deficit hyperactivity disorder, and schizophrenia demonstrate abnormal trial-to-trial neural variability compared to healthy controls (Trenado *et al.*, 2019), this area has been largely underexplored in RewP research.

Finally, the current study expands on previous research examining group-level internal consistency estimates of RewP. Group-level estimates indicate whether between-person variance (i.e. differences between person average scores) is larger than the average between-trial variance (i.e. differences between trial scores within a person), justifying subsequent analysis of individual differences (e.g. relationship with other correlates). Most ERP studies have calculated reliability using two approaches derived from classical test theory. The first is split-half reliability (*r*_xx_), calculated by examining the correlation between two halves of the data, and correcting this estimate using the Spearman–Brown prophecy formula (Nunnally *et al.*, 1967). This approach is beneficial because it includes all trials available for all participants. However, this approach is limited in that it is specific to only one way of splitting the data. Thus, the typical ERP study also calculates Cronbach’s alpha (α; Cronbach, 1951), which is approximately equal to computing all possible split-half correlations. However, this approach also has significant shortcomings. The calculation of Cronbach’s alpha requires an equal number of trials between participants, typically resulting in the exclusion of some participants, trials, or both. In response to some of the limitations of the classical test theory, Clayson, Miller, and colleagues (Clayson and Miller, 2017; Clayson *et al.*, 2021a, 2021b) have proposed that generalizability theory (G theory) may be more suitable for use in ERP research. G theory augments classical test theory estimates by considering multiple sources of variance such as measurement occasion, diagnostic group, number of trials, and event type, in addition to unaccounted for measurement error. Perhaps, the most significant advantage of G theory over classical test theory is that it can handle unbalanced designs (e.g. unequal number of trials retained for averaging), which is common in ERP studies due to artifact rejection procedures necessary for analyzing ERP scores (Clayson and Miller, 2017). In brief, G theory calculations result in two different types of reliability coefficients: (i) generalizability coefficient (*E*ρ^2^_D_) and (ii) dependability coefficient (ϕ)_D_. The generalizability coefficient (*E*ρ^2^) is concerned with relative decisions (whether participants are ranked similarly in each condition of a facet). The dependability coefficient (ϕ) is concerned with absolute decisions (similar scores between conditions of a facet). Given the doors task contains two feedback conditions (i.e. gain and loss feedback) that remain constant across trials and participants (i.e. no variability in valence of reward or loss), the ranking of ERP scores within an object of measurement is not of interest. As such, previous RewP literature has relied on the dependability coefficient to calculate group-level reliability obtained from the doors task (Levinson *et al.*, 2017; Distefano *et al.*, 2018; Ethridge and Weinberg, 2018; Clayson *et al.*, 2021b). Briefly, group-level dependability (ϕ_k_) is estimated as a function of between-person variance (σ*^2^_p_*), between-trial variance (σ*^2^_ik_*), and a given number of trials (*n_ik_*′). The number of trials used for *n_ik_*′ is a central tendency estimate (e.g. mean or median) for the number of included trials for a group of participants. When between-person variance is large compared to error variance, ϕ_k_ will be high. When between-person variance is small compared to error variance, ϕ_k_ will be low. ϕ_k_ will range between zero and one, with estimates closer to one indicating higher levels of dependability (reliability; Clayson *et al.*, 2021b).

In the current study, we examined the psychometric properties of RewP in a large community sample of adult women. We first examined the group- and subject-level internal consistency of RewP. Although we emphasize the utility of G theory to estimate the internal consistency of the RewP, we also report estimates using classical test theory to facilitate the comparison to previous studies. Next, we report the 8-week test–retest reliability of RewP. Consistent with previous literature, we hypothesized that RewP-gain and RewP-loss would demonstrate good-to-excellent internal consistency and test–retest reliability, but ΔRewP would demonstrate low-to-moderate reliability. Next, we report the relationship between depression and averaged RewP scores. We hypothesized that ΔRewP, but not RewP-gain or RewP-loss, would relate to symptoms of depression. Finally, we examined whether age and depression symptoms are potential factors that contribute to poor subject-level reliability.

## Methods

### Participants

Participants comprised 279 adult females who were mothers of adolescents enrolled in a large longitudinal trial examining the effect of attention bias modification (ABM) on psychophysiological measures in a community sample of youth (ClinicalTrials.gov Identifier: NCT03176004).^2^

We used the ERP reliability analysis (ERA) Toolbox v. 0.5.2 to determine the number of trials needed to achieve stable average RewP scores (Clayson and Miller, 2017). We excluded three participants who did not have enough trials to achieve a reliable RewP in either the gain (9 trials) or loss conditions (10 trials) at baseline, resulting in 276 participants. Participants’ ages ranged from 27 to 58 years old (*M* = 44.57, SD = 5.90).^3^ Sixty-two participants were identified as Hispanic or Latino (23.5%). Ethnicity data were not available for one participant. Most participants self-identified as White (*n *= 213, 77.2%), 11 as Black (4.0%), 13 as Asian (4.7%), four as Native Hawaiian or Pacific Islander (1.4%), two as Native American or Alaskan Native (0.72%), 12 as two or more races (4.3%), and 21 declined to answer or did not have race data available (7.6%). Median household income of the sample was $100 000. The distribution of key demographic variables from this sample is provided in the Supplementary Materials. A subset of these participants (*n* = 192) then returned to the lab 8 weeks later to complete the doors task again. We excluded five participants who did not have enough trials needed to achieve a reliable RewP in either the gain (11 trials) or the loss (11 trials) conditions at the 8-week visit. Thus, the matched sample at baseline and 8 weeks comprised 187 participants. Demographic information for the sample at 8 weeks is reported in the Supplementary Materials.

Participants were recruited as part of a larger longitudinal study of ABM in their adolescent offspring. Thus, not all the mothers of the participants in that longitudinal study reported on their own depression. This measure was implemented toward the end of baseline data collection and was administered to participants again at a third visit 2 years later. Thus, 46 participants completed our measure of depression at baseline and 80 more mothers completed this measure during the third visit 2 years later for a total of 126 participants for the validity analysis. This subsample of participants was similar in age to the first visit sample (*M* = 44.98, SD = 5.36).^4^ Thirty-three participants identified as Hispanic or Latino (26.2%). Ethnicity data were not available for one participant. One hundred three participants were identified as White (81.7%), three as Black (2.4%), six as Asian (4.8%), one as Native American or Alaskan Native (0.79%), one as two or more races (0.79%), and 12 declined to answer or did not have race data available (9.5%). Median household income of the sample was $110 000. The distribution of key demographic variables from this sample is provided in the Supplementary Materials.

### Procedure

Participants completed a computerized monetary guessing task while we recorded EEG continuously. We used Presentation Software (Neurobehavioral Systems, Systems, Inc., Albany, CA) to present the task to participants. Participants returned to the laboratory 8 weeks after the first visit to complete the same task again. Some participants returned for a third visit to complete the same computer task as well as a measure of depressive symptoms. We compensated participants at a rate of $20 per hour for their participation. In addition, they received $7.50 for their winnings from the doors task. Participants provided written informed consent. The Institutional Review Board approved all procedure described here.

### Tasks and materials

#### Doors task

In the doors task, participants see two identical images of doors on a computer screen (Foti and Hajcak, 2010). We ask participants to select one of the doors by pressing a mouse button on that door. The winning door results in a monetary reward of $0.50, represented by a green arrow pointing up, while selecting the losing door results in losing $0.25, represented by a red arrow pointing down. We used this ratio as monetary losses are twice as valuable as monetary gains (Tversky and Kahneman, 1992). The experiment comprised 60 trials divided into 3 blocks of 20 trials each. The blocks were separated by participant-timed breaks, during which the instructions ‘Pause – Click when ready to continue’ appeared on the screen until the participant clicked. Unbeknownst to the participants, there were an equal number of wins and loss trials (i.e. 30 each), regardless of the doors selected. Following a brief description of the experiment, we attached EEG sensors to the participant scalp and provided them with detailed task instructions.

The sequence and timing of the task stimuli was as follows: We presented a fixation cross (+) in the center of the screen for 500 ms. Next, an image of the two doors appeared until the participants clicked the left or right mouse button. Then, participant saw the fixation cross again for 1500 ms followed by an upward facing green arrow (representing a win trial) or a downward facing red arrow (representing a loss trial) for 2000 ms. Next, participants saw another fixation cross for 1500 ms and the word ‘Click for next round,’ which appeared on the bottom of the screen until the participant clicked either mouse button. The total duration of the task ranged between 5 and 7 min.

#### Beck Depression Inventory-II

The Beck Depression Inventory-II (BDI-II) is one of the most used self-rating questionnaires for measuring the severity of depression in adults. It comprises 21 questions assessing the somatic, cognitive, and affective symptoms of depression, with the items rated on four-point scales ranging from 0 to 3 with a maximum total score of 63 (higher scores indicate severe depressive symptoms; Beck *et al.*, 1996). The BDI-II demonstrates excellent internal consistency (α = 0.83–0.96), test–retest reliability (*r *= 0.73–0.96), and concurrent validity (Wang and Gorenstein, 2013). In the current sample, the BDI-II showed excellent internal consistency (α = 0.88). The BDI-II comprises a two-factor scoring: cognitive and noncognitive symptoms. These two factors have both theoretical (Beck *et al.*, 1996) and factor analytic support (Dozois *et al.*, 1998; Steer *et al.*, 1999, 2000; Viljoen *et al.*, 2003). The BDI-II cognitive items comprise general depressive symptoms, whereas noncognitive items comprise somatic symptoms and anhedonia symptoms of depression.

### Psychophysiological recording, data reduction, and analysis

We followed recommendations from Keil *et al.* (2014) to provide all information on data collection and preprocessing. We collected EEG data using a 32-electrode Brainvision ActiChamp system (Brain Vision ActiChamp System, 2016) and an EasyCap electrode cap (EasyCap GmbH, n.d.). The active electrodes recorded (FP1, Fz, FCz, F3, F7, FC5, FC1, C3, T7, TP9, CP5, CP1, CPz, Pz, P3, P7, O1, Oz, O2, P4, P8, TP10, CP6, CP2, Cz, C4, T8, FC6, FC2, F4, F8, and FP2) were a subset of the international 10–20 system. We sampled the data at 1000 Hz utilizing a low-pass online filter set at 100 Hz and referenced all channels to Cz during data collection. The ground electrode was placed at FPz. We used vertical electrooculogram (EOG) passive electrodes placed above and below the left eye to detect eyeblinks and horizontal EOG electrodes on either temple to detect lateral eye movement, with ground placed on the forehead.

We performed all offline data processing in MATLAB (MathWorks Inc., Massachusetts, USA), the EEGLAB open source toolbox (Delorme and Makeig, 2004), and the ERPLAB toolbox (Lopez-Calderon and Luck, 2014). We re-referenced the data to the average of the mastoid electrodes (M1 and M2) and removed noisy channels via the Artifact Subspace Reconstruction method by using the clean_artifacts function within the clean_rawdata plugin in EEGLAB. Those channels were interpolated using a spherical interpolation algorithm. We did not interpolate channels used for referencing or for measurement (i.e. M1, M2, Fz, FCz, and Cz). A second-order infinite impulse response Butterworth filter was used for bandpass filtering on the continuous (nonsegmented) data between 0.01 and 30 Hz with a 12 dB/octave roll-off. We created epochs from −200 to 800 surrounding the presentation of the feedback stimulus [green up arrow (win) or red down arrow (loss)]. Data were baseline corrected between −200 and 0 ms prior to the response. We implemented a semiautomatic artifact rejection and correction such that trials containing less than −200 μV or greater than 200 μV were removed. Next, we used a Moving Window Peak to Peak trial rejection to remove trials with a threshold difference greater than 150 μV in a 500 ms window and 100 ms window step. Next, we corrected for vertical and horizontal EOG artifacts using the method developed by Gratton and colleagues (Gratton *et al.*, 1983; Miller *et al.*, 1988). Finally, we quantified the RewP as the mean amplitude in the EEG signal in the time window 200 to 350 ms at FCz electrode site, where the ΔRewP was maximal for our sample. We quantified ERP values separately following the presentation of the reward (RewP-gain) and loss (RewP-loss) feedback. The ΔRewP was quantified as the mean difference between gain and loss trials (RewP-gain minus RewP-loss).

### Data analytical plan

We conducted all statistical analyses in the R (version 4.1.0) programing language. Experimental effects of the task were assessed using a pairwise *t*-test between the gain and loss conditions. We calculate the dependability coefficient (ϕ) from G theory. Phi represents an estimate of internal consistency that is analogous to coefficient alpha from classical test theory (Shavelson and Webb, 1991). We used the ERA Toolbox v 0.5.2 (Clayson and Miller, 2017) in MATLAB (version, 2019b) to calculate RewP score dependability based on algorithms from G theory and used CmdStan v 2.19.0 (Stan Development Team, 2019 to implement the analyses in Stan (Carpenter *et al.*, 2017). To estimate the internal consistency of the ΔRewP, we used equations from classical test theory and generalizability theory (Clayson *et al.*, 2021a). Finally, we assessed 8-week test–retest stability of each ERP measure using Pearson’s correlations and dependability coefficients of stability from G theory (Clayson *et al.*, 2021c). To establish criterion validity, we examined the continuous relationship between RewP measures and depressive symptoms as measured by the BDI-II.

## Results

### Descriptive statistics

**Fig. 1.**
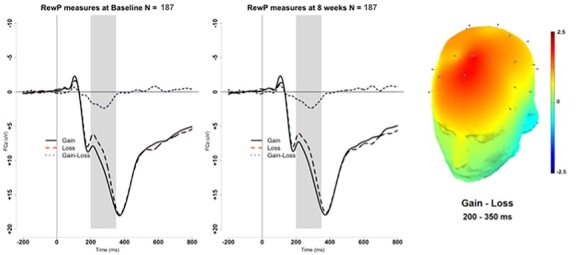
RewP measures at FCz at baseline (left) and 8 weeks (right). The scalp topography reflects average topography for the matched sample at baseline (*N* = 187) for ΔRewP (Gain–Loss) between 200 and 350 ms.

Descriptive statistics for the matched sample at baseline and 8 weeks are displayed in Table 1. Figure 1 presents the grand-average stimulus-locked ERPs at FCz for RewP-gain, RewP-loss, and ΔRewP at baseline and 8 weeks. The topographical scalp plot of the ΔRewP at baseline is also presented in Figure 1.

**Table 1.** Mean (SD) of RewP measures at baseline (*N* = 276) and 8 weeks (*N* = 187)

	Time 1, mean (SD)	Time 2, mean (SD)
RewP-gain	11.64 (6.35)	11.05 (5.98)
RewP-loss	9.87 (5.89)	9.24 (5.82)
ΔRewP (gain–loss)	1.77 (2.82)	1.81 (2.95)

### Doors experimental effects

To establish basic experimental effects of the doors task, we conducted pairwise *t*-tests between conditions. Results revealed that the RewP-gain was larger than the RewP-loss at baseline [*t*(275) = 10.44, *P* < 0.001, 95% CI (1.44, 2.11), Cohen’s *d_z_ = *0.63]. This effect was replicated at 8 weeks such that the RewP-gain was larger than RewP-loss [*t*(185) = 8.28, *P* < 0.001, 95% CI (1.35, 2.19), Cohen’s *d_z_ = *0.61].

### Data quality

**Fig. 2.**
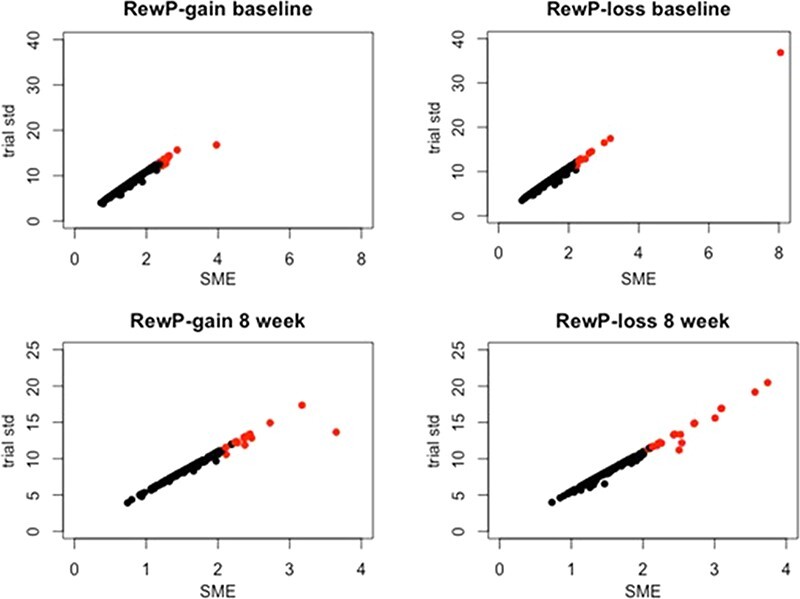
Relationship between data quality estimates for RewP-gain and RewP-loss at baseline (*N* = 276) and 8 weeks (*N* = 187).

Note: SME = standardized measurement error; Trial std = between-trial standard deviation. Participants with poor subject-level dependability (see Section 3.4) are highlighted in red. Note different limits on *x*- and *y*-axes.

The two data quality estimates of interest, SME (SME*_ij_*), and between-trial standard deviations (σ*_ij_*) are reported in Table 2 for baseline and 8 weeks. Figure 2 reveals a strong linear relationship between SME and between-trial standard deviations. This is expected when the number of trials included in an estimate is very similar across persons, as is the case for RewP (Clayson *et al.*, 2021c). As Luck *et al.* (2021) pointed out, it is difficult to know what constitutes a ‘small enough’ SME score, but comparing SME estimates among participants can shed light on which participants might have poor data quality relative to other participants within a group. Visual inspection of the baseline (top left and right) panels for RewP-gain and RewP-loss suggests one participant has poor data quality (high SME and between-trial standard deviation) relative to other participants. Corroborating this conclusion, the same participant had poor data quality for both the gain and loss conditions, suggesting poor data quality overall for that participant. As shown in both the 8-week (bottom left and right) panels for RewP-gain and RewP-loss, it is less clear which participants have poor data quality relative to the rest of the sample. In order to explore the data quality further, participants who also demonstrate poor subject-level dependability (see Section 3.4) are colored in red. These measures may be used in conjunction to determine which participants are not representative of the whole sample. However, because subject-level dependability clarifies whether person-specific data quality is high enough within a specific group and provides a clear ‘cutoff’ score, subject-level dependability may be a more methodical measure to determine which individuals are not characteristic of the rest of the sample.

**Table 2.** Summary Statistics for ERP to Gain and Loss Trials

	Gain		Loss	
Measurement	M (SD)	Range	M (SD)	Range
*No. of trials*				
Baseline	29.36 (1.64)	18–30	29.32 (1.68)	19–30
Eight weeks	29.47 (1.51)	14–30	29.43 (1.51)	20–30
*Data quality*				
SME*_ij_*				
Baseline	1.53 (0.41)	0.73–3.95	1.48 (0.55)	0.66–8.05
Eight weeks	1.60 (0.46)	0.74–4.24	1.56 (0.47)	0.73–3.74
σ*_ij_*				
Baseline	8.25 (2.14)	3.80–16.77	7.99 (2.69)	3.45–36.87
Eight weeks	8.65 (2.35)	3.91–22.83	8.44 (2.52)	3.99–20.48
*Group-level internal consistency*				
	Estimate	95% CI	Estimate	95% CI
*r*_xx_				
Baseline	0.95	—	0.93	—
Eight weeks	0.93	—	0.93	—
α				
Baseline	0.94	(0.93, 0.95)	0.94	(0.93, 0.95)
Eight weeks	0.93	(0.92, 0.95)	0.93	(0.92, 0.95)
ϕ*_k_*				
Baseline	0.93	(0.92, 0.94)	0.93	(0.92, 0.94)
Eight weeks	0.93	(0.91, 0.94)	0.93	(0.91, 0.94)
ICC				
Baseline	0.32	(0.28, 0.36)	0.30	(0.27, 0.34)
Eight weeks	0.28	(0.23, 0.32)	0.27	(0.23, 0.32)
*Subject-level internal consistency*				
	M (SD)	Range	M (SD)	Range
ϕ*_jk_*				
Baseline	0.93 (0.02)	0.81–0.96	0.93 (0.03)	0.58–0.96
Eight weeks	0.92 (0.03)	0.77–0.97	0.92 (0.03)	0.79–0.97
ICC				
Baseline	0.33 (0.06)	0.18–0.49	0.32 (0.06)	0.06–0.46
Eight weeks	0.30 (0.06)	0.10–0.48	0.30 (0.06)	0.11–0.48

*Note:* All estimates based on *N* = 276 at baseline and *N* = 187 at 8 weeks. SME_ij_ = standard measurement error; σ_ij_ = between-trial standard deviation; *r*_xx_ = odd–even reliability with Spearman–Brown Prophecy adjustment; α = Cronbach’s alpha; ϕ_k_ = group-level dependability; ϕ_jk_ = subject-level dependability.

### Subject-level internal consistency

**Fig. 3.**
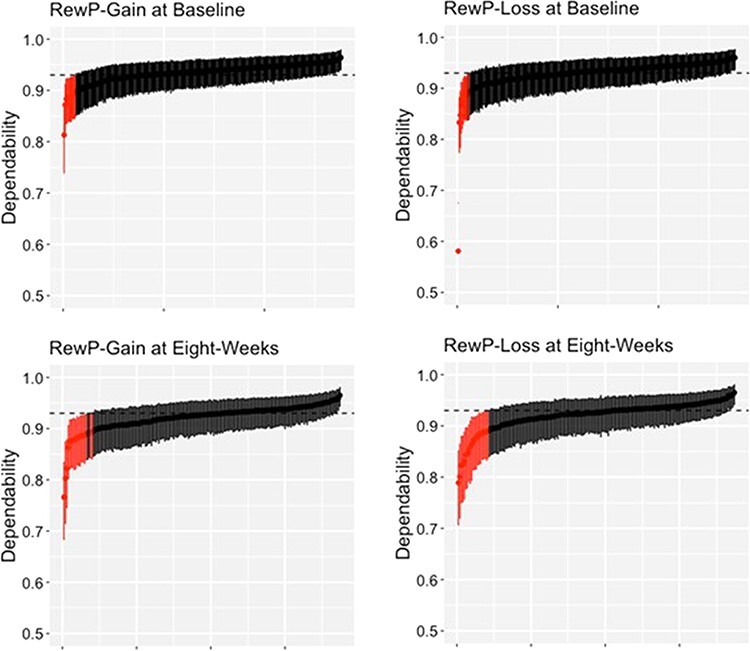
Subject-level dependability estimates (ϕ*_jk_*) for each person with their respective 95% credible intervals.

Note: When the credible intervals do not include the group-level dependability estimate (shown on the dotted line), the credible intervals are highlighted in red. Data are ordered from the smallest to largest ϕ_jk_ estimate.

**Fig. 4.**
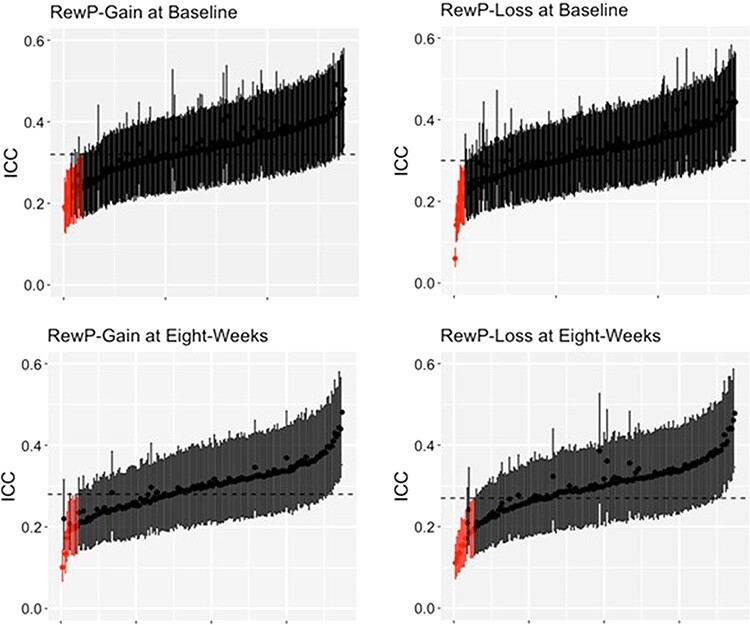
Subject-level intraclass correlation coefficients (ICC*_jk_*) for each person with their respective 95% credible intervals.

Note: When the credible intervals do not include the group-level ICC (shown on the dotted line), the credible intervals are highlighted in red. Data are ordered from the smallest to largest ICC_jk_ estimate.

Summary statistics for subject-level dependability and intraclass correlation coefficients (ICCs) are summarized in Table 2. Individual subject-level dependability and ICCs are plotted in Figures 3 and 4. Data for participants with Bayesian 95% confidence interval (a.k.a., credible interval) that do not include the group-level estimate are highlighted in red. These plots provide a simple visualization of how well group-level internal consistency characterizes individual participant data. At baseline, the group-level dependability estimates were not reached by 12 (4.35%) and 11 (3.99%) participants in the gain and loss conditions, respectively (see Figure 3). At baseline, the group-level ICC estimates were not reached by 13 (4.71%) and 10 (3.62%) participants in the gain and loss conditions, respectively (see Figure 4). At 8 weeks, the group-level dependability estimates were not reached by 18 (9.63%) and 21 (11.23%) participants in the gain and loss conditions, respectively (see Figure 3). At 8 weeks, the group-level ICC estimates were not reached by 8 (4.28%) and 11 (5.88%) participants in the gain and loss conditions, respectively (see Figure 4).

### Group-level internal consistency

Acceptable values of alpha range from 0.70 to 0.95 (Nunnally and Bernstein, 1994; Bland and Altman, 1997; Tavakol and Dennick, 2011; DeVellis, 2017). Clayson and Miller (2017) recommended an internal consistency threshold (dependability, Cronbach’s α, split-half, etc.) of 0.80 for ERP studies. Classical test theory–derived measures (Cronbach’s α and split-half) showed that RewP-gain and RewP-loss showed excellent internal consistency (0.93–0.95) at baseline and 8 weeks (see Table 2). As Cronbach’s alpha requires all participants to have the same number of trials, we computed alpha using the minimum number of trials available for all participants at baseline: RewP-gain (18) and RewP-loss (19) and at 8 weeks: RewP-gain (14) and RewP-loss (20). Similarly, G theory–derived dependability estimates for RewP-gain and RewP-loss were excellent (0.93; see Table 2) when using all trials retained for each participant. At baseline, minimum recommended dependability scores of 0.80 or above were reached at 9 and 10 trials for RewP-gain and RewP-loss, respectively. At 8 weeks, 11 trials were needed for both RewP-gain and RewP-loss. As mentioned in Section 2.1, three participants at baseline and five subjects at 8 weeks were excluded from subsequent data analysis for not meeting the trial cutoffs required for acceptable (>0.80) group-level dependability (Table 3).

**Table 3.** Mean (SD) of RewP and BDI-II measures (*N* = 126)

	Mean (SD)
RewP-gain	10.60 (6.91)
RewP-loss	9.25 (6.32)
ΔRewP (gain–loss)	1.35 (3.28)
BDI-II cognitive	2.36 (2.85)
BDI-II noncognitive	4.94 (4.28)
BDI-II Total	7.29 (6.46)

To calculate the internal consistency of the ΔRewP, we used equations suggested by Clayson *et al.* (2021a). Consistent with previous literature and as expected, ΔRewP showed low-to-moderate internal consistency using classical test theory estimates at baseline (ρ_DD_′ = 0.38) and 8 weeks (ρ_DD_′ = 0.42). Similarly, ΔRewP showed low internal consistency when using G theory estimates at baseline [ϕ = 0.27, 95% CI (0.16, 0.39)] and 8 weeks [ϕ = 0.31, 95% CI (0.19, 0.44)]. Lower ΔRewP reliability has been attributed to a high correlation between RewP-gain and RewP-loss, which may be the case in the current sample: RewP-gain and RewP-loss were highly correlated at baseline [*r*(274) = 0.90, *P* < 0.001] and 8 weeks [*r*(185) = 0.88, *P* < 0.001].

### Test–retest reliability

Consistent with previous test–retest reliability studies in children and adults (Bress *et al.*, 2015; Levinson *et al.*, 2017; Kujawa *et al.*, 2018), we found good test–retest reliability for RewP-gain [*r*(185) = 0.79, *P* < 0.001, 95% CI (0.73, 0.84)] and RewP-loss [*r*(185) = 0.81, *P* < 0.001, 95% CI (0.75, 0.85)] when using classical test theory. As expected, the ΔRewP demonstrated lower reliability [*r*(185) = 0.37, *P* < 0.001, 95% CI (0.24, 0.49)] than its components. We also calculated a dependability coefficient of stability from G theory, which is analogous to test–retest reliability estimates (Clayson *et al.*, 2021c). We used a dependability cutoff score of 0.80. The number of trials from each session needed was 24 for gain trials and 27 for loss trials. Fifteen participants were excluded after applying the trial cutoffs, resulting in 172 participants with an average of 29.68 gain trials and 29.71 loss trials. We found good stability for RewP-gain [0.78, 95% CI (0.72, 0.83)] and RewP-loss [0.81, 95% CI (0.75, 0.85)] over 8 weeks. The ERA Toolbox does not currently allow for estimation of coefficients of stability for difference scores (Clayson *et al.*, 2021c).

### Relationship between depression and averaged RewP scores

Depression comprises absence of pleasure in situations that would call for it (i.e. anhedonia) or the presence of displeasure (i.e. dysphoria emotions; Berridge and Kringelbach, 2015). A blunted ΔRewP has been associated with anhedonia (Santopetro *et al.,* 2021), as well as general dysphoria symptoms (Distefano *et al.*, 2018; Nelson and Jarcho, 2021). However, there is little evidence for a relationship between somatic symptoms of depression (i.e. sleep disturbances, weight change, and fatigue) and ΔRewP. In the current paper, we examined the relationship between RewP measures separately for cognitive items (i.e. sadness, pessimism, past failure, guilty feelings, punishment feelings, self-dislike, self-criticalness, suicidal thoughts or wishes, indecisiveness, and worthlessness) and noncognitive items (i.e. loss of pleasure, crying, agitation, loss of interest, loss of energy, changes in sleeping pattern, irritability, changes in appetite, concentration difficulty, tiredness or fatigue, loss of interest in sex) of the BDI-II. Both the BDI-II cognitive (*a* = 0.79) and noncognitive (*a* = 0.83) item subscales demonstrated good internal consistency.

Results are presented in Table 4. BDI-II cognitive scores were significantly correlated with ΔRewP, such that higher depressive symptoms were related to a blunted ΔRewP. BDI-II cognitive scores were not significantly correlated to RewP-loss or RewP-gain. BDI-II total scores and BDI-II noncognitive scores were not significantly correlated with average RewP measures. The correlation between BDI-II cognitive scores and ΔRewP was no longer significant when we controlled for the false discovery rate (FDR) using the method of Benjamini and Hochberg (Benjamini and Hochberg, 1995). We considered a family, scores within a class of measures (i.e. depression or dependability) when correlated with another class.

**Table 4.** Correlations between RewP, subject-level reliability, BDI-II scores, and age (*N* = 126)

	1	2	3	4	5	6	7	8
1. BDI-II Total								
2. BDI-II cognitive	0.86*							
3. BDI-II noncognitive	0.94*	0.63*						
4. Age^a^	−0.15	−0.10	−0.17					
5. Depend RewP-gain	−0.20^**^	−0.17	−0.19^**^	0.10				
6. Depend RewP-loss	−0.14	−0.10	−0.15	0.08	0.79*			
7. RewP-gain	−0.09	−0.16	−0.03	−0.05	−0.01	0.18^**^		
8. RewP-loss	−0.05	−0.08	−0.02	−0.04	−0.09	0.08	0.88*	
9. Delta RewP	−0.10	−0.18^**^	−0.03	−0.02	0.14	0.22^**^	0.41*	−0.07

*Note:* *P < 0.001;

** *P* < 0.05;

a Age data were not available for five participants. Only the correlations within a family (i.e. BDI scores or dependability scores) survived the false discovery rate correction.

We repeated reliability and data quality analyses for this subsample of participants (see Supplementary Materials for results). 11 participants demonstrated poor subject-level reliability for gain or loss trials. After removing these participants, the relationship between ΔRewP and BDI-II cognitive scores was only marginally significant [*r*(113) = -0.17, *P* = 0.08, 95% CI (−0.34, −0.02)]. All other correlations between BDI-II measures and average RewP measures remained nonsignificant (*P*’s >0.05).

### Relationship between subject-level dependability, age, and depression

To determine whether age or depressive symptoms contribute to poor subject-level reliability, we performed correlational analyses between subject-level dependability, age, and BDI-II total and subscale scores. Results are presented in Table 4. BDI-II total and BDI-II noncognitive scores were significantly correlated with RewP-gain dependability, such that higher depressive symptoms were related to poorer subject-level reliability on reward trials. However, this correlation was not significant after controlling for the FDR using the method of Benjamini and Hochberg method (Benjamini and Hochberg, 1995). Age was not significantly correlated to any of our measures of depression or subject-level dependability. After removing the 11 participants who demonstrated poor subject-level reliability for gain or loss trials, the relationship between RewP-gain dependability and BDI-II total scores was no longer significant [*r*(113) = −0.14, *P* = 0.15, 95% CI (−0.31, −0.04)]. All other correlations between RewP-gain dependability and RewP-loss dependability and average remaining measures remained nonsignificant (*P*’s > 0.05).

## Discussion

In the present study, we examined the psychometric properties of the RewP in a large adult sample using both generalizability and classic test theory constructions of reliability. Consistent with previous research in adults (Levinson *et al.*, 2017; Ethridge and Weinberg, 2018), we found that both the RewP-gain and RewP-loss demonstrated excellent group-level internal consistency based on split-half reliability, Cronbach’s alpha, and G theory estimates of dependability. We also calculated subject-level internal consistency estimates at baseline and 8 weeks using dependability estimates, finding that most participants demonstrated good-to-excellent subject-level reliability. While group-level estimates of internal consistency were representative of the majority of our sample, these estimates mischaracterized between 3 and 11% of participants, suggesting that subject-level internal consistency provides additional information that focuses on individual differences in RewP measures. It is possible that participants with lower subject-level reliability may contribute to misinterpretation or non-replicable results at the group level. This is particularly important in studies that examine the relationship between the RewP and psychopathology. We found this to be the case in the current study. We examined the relationship between reward processing and depression by correlating a dimensional measure of depressive symptoms with RewP measures, finding that a smaller ΔRewP is related to increased symptoms of depression. Specifically, the ΔRewP related to cognitive symptoms of depression, as opposed to a subscale comprising somatic and anhedonic symptoms of depression. This is in line with previous research demonstrating that the ΔRewP relates to subscale measures of anhedonia (Santopetro *et al.*, 2021) and general dysphoria symptoms (Distefano *et al.*, 2018; Nelson and Jarcho, 2021) when examined in a community sample. However, after excluding participants with poor subject-level dependability, this correlation was no longer significant. Thus, the ΔRewP only correlated marginally with depressive symptoms in a large community sample of women. Future studies should establish subject-level dependability in their study as a precursor to examining individual differences of RewP measures.

We found that both the RewP-gain and RewP-loss demonstrated good long-term test–retest reliability measured across 8 weeks. This is within the typical timeframe for evidence-based treatment batteries (i.e. 2–4 months). Thus, the RewP may be a useful and reliable measure to predict treatment response in an adult sample (Klawohn *et al.*, 2021). However, these findings should first be replicated in a clinical adult sample. Consistent with previous research in children and adults, the ΔRewP (RewP-gain − RewP-loss) demonstrated lower internal consistency and test–retest reliability (Bress *et al.*, 2015; Levinson *et al.*, 2017; Luking *et al.*, 2017; Ethridge and Weinberg, 2018; Kujawa *et al.*, 2018) than its constituent scores when using both classical test theory and G theory estimates at baseline and 8 weeks (Clayson *et al.*, 2021b). Difference score reliability is impacted by the internal consistency of its constituent scores and the correlation between them (Clayson *et al.*, 2021a; Furr and Bacharach, 2014, Chapter 6). Previous research has suggested that the ΔRewP demonstrates lower reliability when compared to other difference score ERPs, such as the error-related negativity (ΔERN), due to a much higher correlation between RewP-gain and RewP-loss (0.79) than the ΔERN constituent scores (0.28; Clayson *et al.*, 2021a). In the current study, RewP-gain and RewP-loss were highly correlated (0.88–0.90), likely contributing to the low reliability scores. The reliability of a measure indexes how much true score is contained within that measure as opposed to error variance. Thus, the ΔRewP is more constrained in the amount of true score available when compared to its constituent scores. Thus, one reason for the lower reliability of the ΔRewP as compared to its constituent scores is the high correlation between the constituent scores. A second argument for the low reliability of ΔRewP is that one would not expect a high test–retest reliability for ΔRewP if it varies with transient depressive symptoms. That is, to the extent that depressive symptoms vary over time, so should the ΔRewP. However, if ΔRewP corresponds to stable depressive symptoms, then its test–retest reliability should be in the range of self-report measures of depression. Delta RewP had a higher correlation with depression than its constituent scores. This may indicate that a larger portion of the ΔRewP true score variance relate to individual differences in depression (Levinson *et al.*, 2017). Thus, use of the ΔRewP may be suitable for future examination of individual differences as its constituent scores show excellent internal consistency and test–retest reliability, and it consistently demonstrates a stronger relationship with measures of depression. These results highlight the utility of RewP measures and point out the importance of examining subject-level internal consistence in clinical setting. Indeed, they call for better standard for acceptable subject-level internal consistency in clinical studies.

We also examined the relationship between RewP subject-level dependability, age, and depression severity. We found that reduced dependability on gain trials was related to higher depression severity; however, our correlational analysis did not survive correction for multiple comparisons, and hence, these results should be viewed with caution. More research is needed to examine whether individuals with depression not only experience overall blunted response to reward (average ΔRewP) but experience increased variability in their response to reward (subject-level dependability). Finally, we did not find a relationship between any of our subject-level dependability estimates and age. Although our study included participants from a wide age range, they were all middle-aged adults. Future studies examining RewP measures in participants across multiple age groups (e.g. children, adolescents, and young adults) should examine this question.

Our study has limitations. For example, our sample included only women. Future research should expand this line of work to include men. We attempted to obtain data from a wide age range, ethnicity, and recruit from a community sample, in order to increase the generalizability of the results. However, examination of other factors, such as gender, socioeconomical status, and developmental consideration, should be addressed in future studies. We also did not exclude participants using criteria typical of EEG and neuroimaging studies (e.g. no history of previous head trauma or neurological issues). All participants in the current study were included if their adolescent child met this eligibility criteria. Thus, it is possible that some participants have a history of health-related issues. Also, we used a monetary doors task to elicit ERP measures of reward processing. Growing evidence suggest that social reward may influence reward sensitivity differently than monetary rewards (Distefano *et al.*, 2018; Ethridge and Weinberg, 2018; Nelson and Jarcho, 2021) and the comparative clinical utility of monetary versus social rewards should be explored. Finally, one issue with the doors task may be that the outcome (win/loss) is confounded with the outcome symbol (color and orientation) and participants may simply be responding to the symbols and colors and not the actual monetary outcome. This possibility should be examined in future studies.

These limitations notwithstanding, the results of the present study suggest that the reward processing components elicited from the monetary doors task remain relatively stable with predictive utility in assessing self-report symptoms of depression.

## Supplementary Material

nsad007_Supp

## Data Availability

The data and code reported for the study can be obtained from OSF: https://osf.io/mvpxw/.

## Footnotes

1 Previous research has been inconsistent in the naming convention of reward-processing ERPs. Feedback negativity (FN) and RewP have been used to refer to raw ERP measures following reward and loss feedback, as well as the difference between the two. To increase clarity, in this paper, we refer to the raw ERP measures following feedback as RewP-gain and RewP-loss. We will refer to the difference between the two as ΔRewP.

2 As part of the larger study, participants in the current study also completed a Flanker task and emotional-interrupt task. The emotional-interrupt task data is reported here in McGhie *et al.* (2021).

3 Age data were unavailable for five participants at baseline. Household income data were unavailable for 28 participants at baseline.

4 Age data were not available for five participants in this subsample. Income data were not available for 19 participants.

## Public significance statement

This study established that a neural marker of reward processing is internally consistent, reliable, and may be related to symptoms of depression in a large sample of adult women.
