# Supplementary material for: The psychometric properties and clinical utility of neural measures of reward processing
Source: Soc Cogn Affect Neurosci. 2023 Mar 15;18(1):nsad007. doi: 10.1093/scan/nsad007 (PMC10039464; doi:10.1093/scan/nsad007)

[Distribution of Key Demographic Variables at Baseline: 2](#_Toc97297434)

[Distribution of Key Demographic Variables at Eight-weeks: 3](#_Toc97297435)

[Distribution of Key Demographic Variables for Validity Sample: 4](#_Toc97297436)

[Spaghetti Plot showing relationship between RewP measures at baseline and eight-weeks (N=187) 6](#_Toc97297437)

[Reliability estimates for Validity Sample: 7](#_Toc97297438)

[Data Quality 7](#_Toc97297439)

[Figure 1 7](#_Toc97297440)

[Table 1 8](#_Toc97297441)

[Group-level Internal Consistency 9](#_Toc97297442)

[Figure 2: Scatterplots of BDI-II and RewP correlations with full validity sample (n=124) prior to removing outliers 10](#_Toc97297443)

# Distribution of Key Demographic Variables at Baseline:


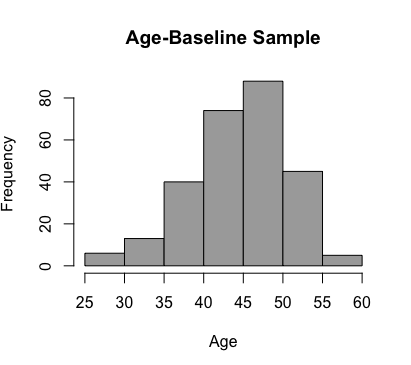

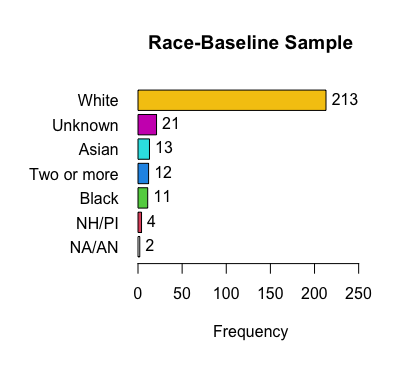


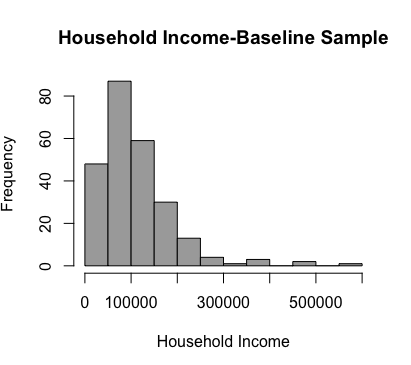


*Note: Based off of N=276 at baseline.*

# Distribution of Key Demographic Variables at Eight-weeks:


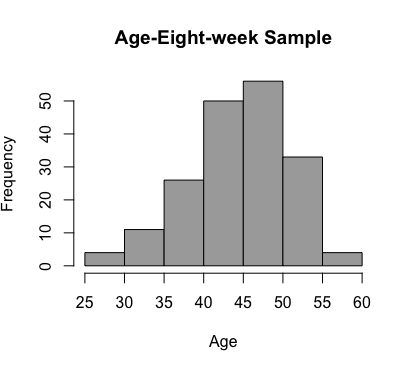

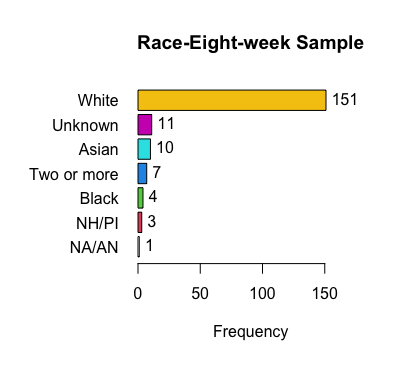


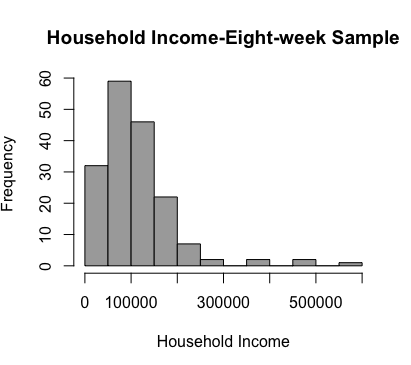


*Note: Based off of N=187 with data at both baseline and eight-week visits.*

# Distribution of Key Demographic Variables for Validity Sample:


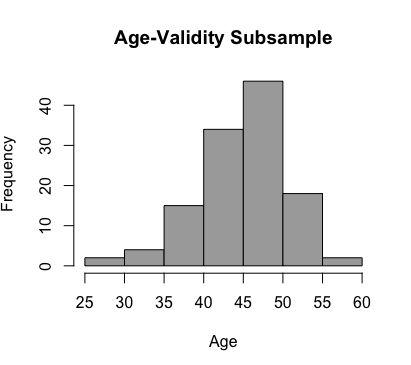

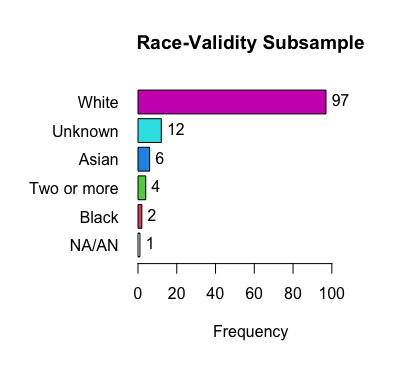


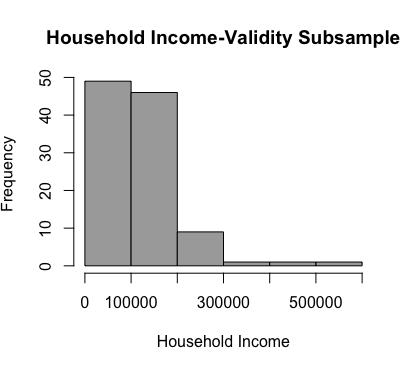


Note: Based on N=126 subsample of participants with both RewP and BDI-II data.

# Spaghetti Plot showing relationship between RewP measures at baseline and eight-weeks (N=187)

**
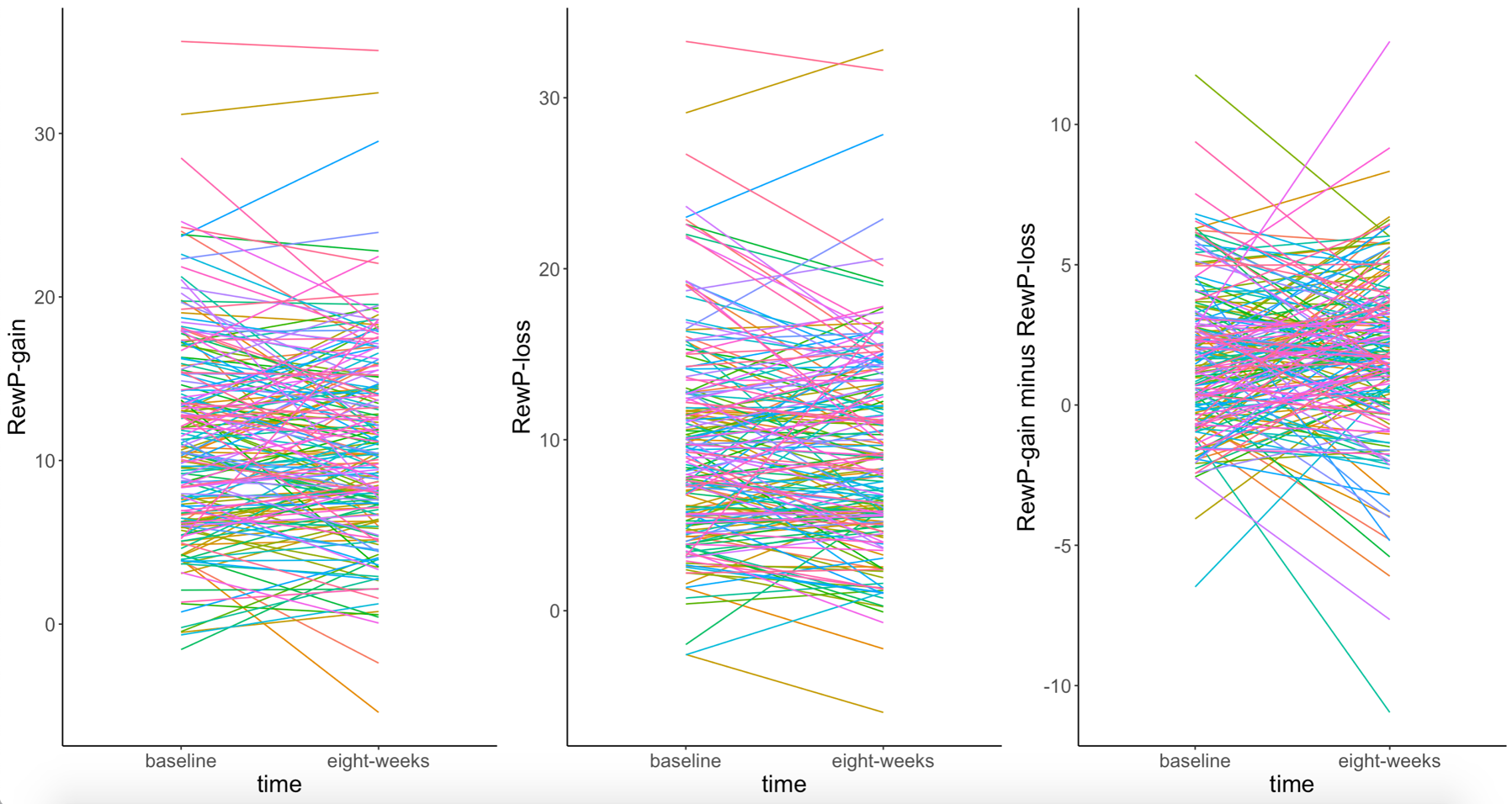
**

# Reliability estimates for Validity Sample:

## Data Quality

The two data quality estimates of interest, SME (*SMEij*) and between-trial standard deviations (*σij*) are reported in Table 1. As acknowledged by Luck et al., 2021, it is difficult to know what constitutes a “small enough” SME score, but comparing SME estimates among participants can shed light on which participants might have poor data quality relative to other participants within a group. Visual inspection of the figues for RewP-gain and RewP-loss suggest two participants had poor data quality (high SME and between-trial standard deviation) relative to other participants for each condition. The same two participants had poor data quality for both the gain and loss conditions. Moreover, both participants demonstrated poor subject-level reliability for the loss condition (<.80; see Section 2). The two participants are shown as red dots in Figure 1. These measures may be used in conjunction to determine which participants are not representative of the whole sample. In the current sample, these two participants were excluded for correlation between RewP and BDI-II scores.

## Figure 1


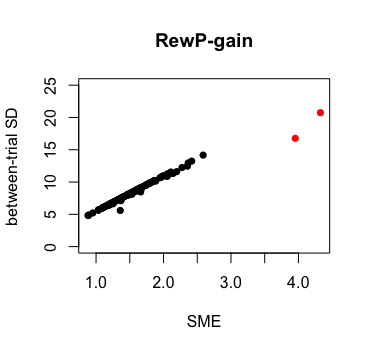

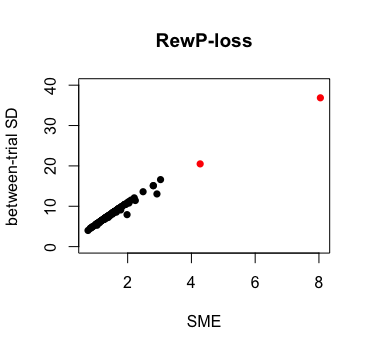


## Table 1

| Measurement | Gain |  | Loss |  |
| --- | --- | --- | --- | --- |
|  | M (SD) | Range | M (SD) | Range |
| # of trials | 29.50 (1.77) | 17-30 | 29.31 (1.95) | 16-30 |
| Data Quality |  |  |  |  |
| *SMEij* | 1.56 (0.48) | 0.88-4.33 | 1.56 (0.76) | 0.76-8.05 |
| *σij* | 8.44 (2.29) | 4.82-20.74 | 8.38 (3.52) | 4.0-36.87 |
| Group-level internal consistency | | |  |  |
|  | Estimate | 95% CI | Estimate | 95% CI |
| rxx | 0.95 | – | 0.93 | – |
| ⍺ | 0.95 | [0.94 0.97] | 0.94 | [0.93 0.95] |
| *ϕk* | 0.95 | [0.94 0.96] | 0.94 | [0.92 0.95] |
| *ICC* | 0.37 | [0.30 0.44] | 0.31 | [0.26 0.37] |
| Subject-level internal consistency | | |  |  |
|  | M (SD) | Range | M (SD) | Range |
| *ϕjk* | 0.94 (0.02) | 0.84-0.97 | 0.93 (0.04) | 0.58-0.97 |
| *ICCjk* | 0.38 (0.05) | 0.18-0.49 | 0.35 (0.08) | 0.06-0.51 |
| *Note:* All estimates based on N = 126. SMEij = Standard Measurement Error; σij = between-trial standard deviation; rxx = odd-even reliability with Spearman-Brown Prophecy adjustment; ⍺ = Cronbach's alpha; ϕk = group-level dependability; ϕjk = subject-level reliability | | | | |

## Group-level Internal Consistency

Classical test theory-derived measures (Cronbach’s α, split-half) showed that RewP-gain and RewP-loss showed excellent internal consistency (>.90). Cronbach’s α was calculated using the minimum number of trials retained for all participants for RewP-gain (17 trials) and RewP-loss (16 trials). Similarly, generalizability theory-derived dependability estimates for RewP-gain and RewP-loss were excellent, when using all trials retained for each participant (See Table 1). Minimum recommended dependability scores of .80 or above were reached at 7 and 9 trials for RewP-gain and RewP-loss, respectively. All participants had enough trials to meet these cut-offs.

To calculate the internal consistency of the ΔRewP, we used equations suggested by Clayson et al. (2021). Consistent with previous literature and as expected, ΔRewP showed lower internal consistency than its constituent scores using classical test-theory estimates (ρDD′ = .38). Similarly, ΔRewP showed lower internal consistency when using generalizability theory (ϕ = .41, 95% CI [0.28, 0.54]). Lower ΔRewP reliability has been attributed to a high correlation between RewP-gain and RewP-loss, which may be the case in the current sample (r(124) = .88, p < .001).

# Figure 2


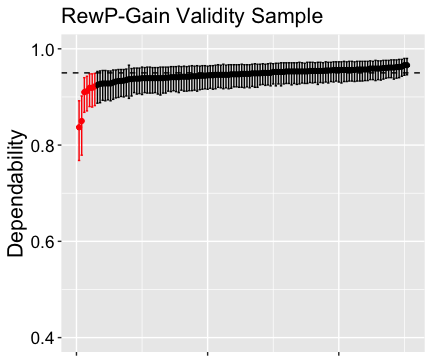

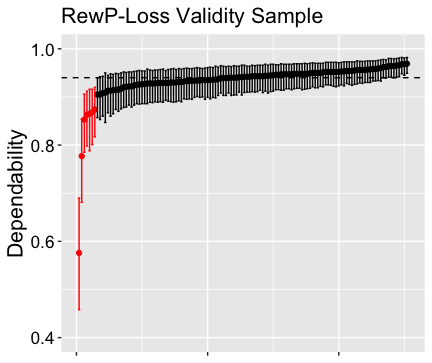


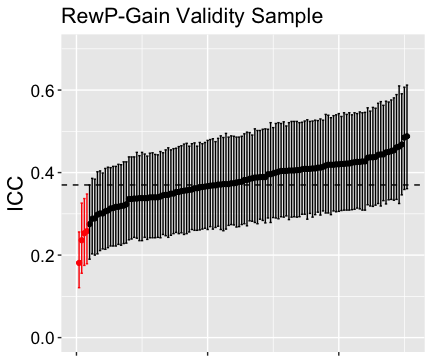

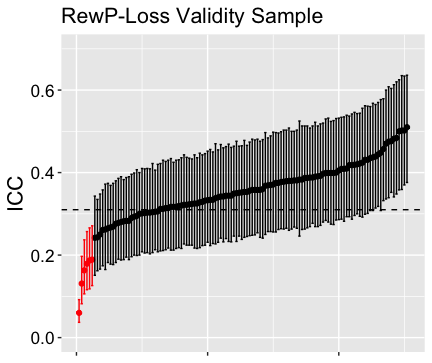

Supplement: nsad007_Supp [file nsad007_supp.zip › scan-21-316-File015.docx]
